# Efficacy and safety of ciprofol versus remimazolam combined with alfentanil for sedation during outpatient colonoscopy: a randomized double-blind noninferiority trial

**DOI:** 10.1016/j.bjane.2026.844824

**Published:** 2026-09-12

**Authors:** Xuan Liu, Qianqian Tang, Ning Zou, Changming Wang, Xuejun Guo

**Affiliations:** a The Dalian Medical University, Department of Anesthesiology, Dalian, China; b People’s Hospital of Liaoning Province (Peoples’ Hospital of China Medical University), Department of Anesthesiology, Shenyang, China; c People’s Hospital of Liaoning Province (Peoples’ Hospital of China Medical University), Department of Neurosurgery, Shenyang, China

**Keywords:** Alfentanil, Ciprofol, Colonoscopy, Conscious Sedation, Gastrointestinal Endoscopy, Remimazolam

## Abstract

**Introduction:**

Colonoscopy often causes significant discomfort to patients. To improve patient experience, sedation-assisted colonoscopy is being increasingly adopted. This study was designed to assess the efficacy and safety of ciprofol versus remimazolam combined with alfentanil during outpatient colonoscopy.

**Methods:**

A randomized, double-blind controlled clinical trial was performed among patients scheduled for elective outpatient colonoscopy. Patients were randomly allocated at a 1:1 ratio to two groups: the ciprofol group (Group C, 0.4 mg.kg^-1^ intravenously, n = 88) and the remimazolam group (Group R, 0.3 mg.kg^-1^ intravenously, n = 88). Intravenous alfentanil (3 μg.kg^-1^) was administered to all patients in conjunction with the study drug. The primary outcome was the success rate of colonoscopy. Secondary outcomes included induction time, discharge time, satisfaction levels (patients and endoscopists), and adverse events.

**Results:**

In the ITT population, colonoscopy success rates were 100.0% (88/88) in Group C and 98.9% (87/88) in Group R (treatment difference [95% CI]: 0.011 [-0.024 to 0.048]). The lower bound of the two-sided 95% CI was above the prespecified noninferiority margin of -0.08, confirming the noninferiority of ciprofol relative to remimazolam. After Bonferroni correction (adjusted threshold p < 0.0028), discharge time was longer in Group C (8.5 [6.0‒12.0] vs. 7.0 [5.0‒9.0] minutes; p = 0.002). Group R showed numerically higher MAP and HR at several time points.

**Conclusions:**

This study demonstrated that intravenous ciprofol (0.4 mg.kg^-1^) or remimazolam (0.3 mg.kg^-1^), combined with alfentanil (3 μg.kg^-1^), provided effective sedation for outpatient colonoscopy.

## Introduction

Colonoscopy is considered an effective primary screening tool for colorectal cancer, but the procedure often causes discomfort in patients.^1^ Therefore, sedation and analgesia are typically required to alleviate these symptoms.^2^^,^^3^ Propofol has been widely used for sedation during colonoscopy. However, its use in outpatient settings is limited by several drawbacks, including injection pain, hypoxemia, and potential cardiorespiratory depression.^4^^,^^5^

Ciprofol is a cyclopropyl‑modified propofol analog with high GABA‑A receptor affinity, providing rapid onset, reliable sedation, and a safety profile similar to propofol.^6^ Remimazolam is an ultra‑short‑acting benzodiazepine characterized by fast onset, quick recovery, favorable hemodynamic stability, and reversibility with flumazenil.^7^^,^^8^ Both agents are promising alternatives to propofol for outpatient endoscopic sedation.^9^ However, their clinical advantages and disadvantages differ in daily practice. In recent years, a number of clinical studies have compared ciprofol and remimazolam for endoscopic sedation. These studies consistently demonstrate that both agents provide effective sedation with favorable safety profiles and low rates of adverse events.^10^^,^^11^ Remimazolam is associated with more rapid recovery and better hemodynamic and respiratory stability,^12^ while ciprofol exhibits reliable sedative effects and a lower incidence of injection pain.^10^ Additionally, comparable overall sedation efficacy has been reported, although the two drugs differ in their effects on intraoperative dreaming.^13^ Our previous study demonstrated that intravenous administration of propofol (1.5 mg.kg^-1^) combined with alfentanil (3 μg.kg^-1^) provides effective sedation for sedation-assisted colonoscopy.^14^ Nevertheless, direct head‑to‑head comparisons of ciprofol versus remimazolam, both combined with alfentanil for outpatient colonoscopy, remain scarce.

To address this issue, we conducted a randomized, double-blind clinical trial to evaluate the efficacy and safety of ciprofol versus remimazolam combined with alfentanil for sedation during outpatient colonoscopy.

## Methods

This double-blind, randomized, controlled study enrolled 176 patients undergoing colonoscopy at our hospital between December 2023 and June 2024. The study protocol was approved by the hospital’s Ethics Committee in September 2022 (approval number: 2022 H002). All participating patients provided written informed consent. The study was prospectively registered at the Chinese Clinical Trial Registry on April 28, 2023 (Registration number: ChiCTR2300070996; URL: https://www.chictr.org.cn). This manuscript was prepared in accordance with the Consolidated Standards of Reporting Trials (CONSORT) guidelines.^15^

Patients aged 18‒65 years with American Society of Anesthesiologists (ASA) physical status I to stable III and a body mass index (BMI) of 18.0‒30.0 kg.m^-2^ scheduled for elective colonoscopy were included. Exclusion criteria were as follows: BMI > 30 kg.m^-2^; expected colonoscopy duration > 30-min; uncontrolled or decompensated severe cardiac, cerebral, pulmonary, hepatic, or renal disease; severe uncontrolled hypertension; preoperative Heart Rate (HR) < 50 beats per minute (bpm) or > 100 bpm; anemia; history of narcotic or sedative abuse; known or anticipated difficult airway; allergy to study medications; pregnancy or lactation; inability to cooperate or communicate; and refusal to participate.

Eligible patients were randomly assigned in a 1:1 ratio to the ciprofol group (Group C, 0.4 mg.kg^-1^ intravenously) and the remimazolam group (Group R, 0.3 mg.kg^-1^ intravenously). A randomization sequence was generated using Statistical Package for Social Sciences (SPSS) software version 27.0 (Inc., Chicago, Illinois, USA) (fixed value: 2000000) by an independent investigator not involved in patient enrollment or clinical care. Patients were enrolled consecutively and allocated to groups in strict chronological order according to this pre‑generated random list. The randomization numbers were encoded, printed on opaque paper, and sealed in sequentially numbered, identical opaque envelopes to ensure allocation concealment.

On the day of the procedure, the envelopes were opened in order by the attending anesthesiologist, who administered the study drugs according to the allocated group. Both study medications were prepared in identical syringes with standardized total injection volume, and syringes were covered with opaque paper during administration. Patients were positioned in the lateral decubitus position with venous access concealed, so they remained blinded to group assignment. The examination area was separated by an opaque curtain. A dedicated anesthesiology assistant, fully blinded to group allocation, monitored vital signs, assessed sedation depth, documented all study outcomes and adverse events, and completed postoperative follow-up assessments. Therefore, the subjects, outcome assessors, endoscopists, and data analysts were blinded to group assignment.

Following premedication with intravenous alfentanil (3 μg.kg^-1^, Yichang Renfu Pharmaceutical Co., Ltd.) given over 30 seconds, sedation was induced after 1-minute with either ciprofol (0.4 mg.kg^-1^, Liaoning Haisho Pharmaceutical Co., Ltd.) in Group C or remimazolam (0.3 mg.kg^-1^, Yichang Renfu Pharmaceutical Co., Ltd.) in Group R.^14^^,^^16^ Sedation depth was assessed with the Modified Observer’s Assessment of Alertness/Sedation (MOAA/S) scale, a validated 6-point scale (0‒5): score 5, responds readily to name spoken in a normal tone; score 4, lethargic response to name spoken in a normal tone; score 3, responds only after name is called loudly and/or repeatedly; score 2, responds only to mild prodding or shaking; score 1, responds only to painful trapezius squeeze; score 0, no response to painful trapezius squeeze. Colonoscopy was initiated when the MOAA/S score was ≤ 2.^17^ All procedures were conducted by experienced endoscopists using an Olympus CV-290 system and CF-H290I colonoscopes. In the event that the MOAA/S score remained > 3 following the initial dose, a supplementary dose (0.1 mg.kg^-1^ ciprofol for Group C or 0.05 mg.kg^-1^ remimazolam for Group R) was permitted after a 1-minute interval. Should this prove insufficient, 0.5 mg.kg^-1^ propofol was to be used as the rescue medication, and study participation was terminated thereafter. Sedation was maintained by assessing the MOAA/S score every 5 minutes and administering supplemental doses as required. Intraoperative adverse events were recorded. Intraoperative hypotension was defined as a MAP < 60 mmHg or an SBP reduction > 30% from baseline and was treated with an intravenous bolus of 20 μg phenylephrine per episode. Bradycardia was defined as a HR < 40 bpm during sedation, representing clinically significant severe bradycardia that requires prompt intervention in gastrointestinal endoscopy settings.^18^ Our management approach is to immediately pause the procedure and administer 0.5 mg atropine intravenously. Furthermore, a sustained SpO_2_ between 75% and 90% for over 60 seconds prompted interventions such as jaw thrust, airway repositioning, or increased oxygen supplementation. If SpO_2_ fell below 75% or apnea persisted beyond 60 seconds, positive-pressure ventilation via face mask or endotracheal intubation was instituted. Upon procedure completion, patients were moved to the Post-Anesthesia Care Unit (PACU). The Post‑Anesthetic Discharge Scoring System (PADSS) was used to assess discharge readiness after colonoscopy sedation. The scale includes five items: 1) Stable vital signs with systolic blood pressure and heart rate within 20% of baseline values; 2) Stable ambulatory ability with independent standing and walking; 3) Fully recovered mental status with clear consciousness and oriented response; 4) Well‑controlled pain with tolerable discomfort; 5) No obvious gastrointestinal bleeding or procedural bleeding. Discharge required a PADSS score ≥ 9 along with stable vital signs.^19^ The quality of recovery within seven days of surgery was assessed by the Quality of Recovery-15‌ (QoR-15) questionnaire.^20^ The QoR‑15 is a validated 15‑item questionnaire assessing postoperative recovery, with higher scores indicating better recovery.

The primary outcome was to evaluate the colonoscopy success rate, which must meet the following criteria: 1) The completion of colonoscopy procedure (colonoscopy can sequentially and clearly observe the status of anal canal, rectum, sigmoid colon, colon and ileocecal mucosa); 2) No need for any alternative/rescue sedative medications; 3) The maximum number of subsequent additions should not exceed 5 doses and should be administered within 15 minutes.^21^ Secondary outcomes included: 1) Induction time (the time from administration to the moment of MOAA/S ≤ 2); 2) Operation time (the time from the insertion of colonoscopy to the withdrawal of colonoscopy); 3) Alert time (the time from the last administration of sedative drugs to the moment of MOAA/S > 4); 4) Discharge time (the time from the end of procedure to the moment of the PADSS score ≥ 9); 5) Patient and endoscopist satisfaction: Patient satisfaction with sedation experience and procedural tolerance, and endoscopist satisfaction with overall sedation quality and procedural conditions, were evaluated using a structured questionnaire. Each assessment was scored on a 10-point scale, with higher scores indicating greater satisfaction; the total score was derived from the sum of individual items;^22^ 6) Use of an improved Brice interview questionnaire to assess intraoperative awareness;^2^ 7) Systolic Blood Pressure (SBP), Diastolic Blood Pressure (DBP), Mean Arterial Pressure (MAP), HR, and Pulse Oxygen Saturation (SpO_2_) were monitored and recorded at the following time points: T1, upon entering the unit; T2, 1 minute after receiving an intravenous injection of ciprofol or remimazolam; T3, colonoscope reaching the ileocecal valve; T4, at the end of the procedure; T5, when leaving the room.

Two analytical populations were defined: the Intention-To-Treat (ITT) population included all randomized patients, regardless of protocol deviations; the Per-Protocol (PP) population was defined as patients who completed the study according to the study protocol, which required completion of the prespecified study procedures and follow-up assessments. Patients who were lost to follow-up, withdrew informed consent, or did not complete the prespecified study procedures and follow-up were excluded from the PP population. In the event of sedation failure, rescue propofol was administered according to the prespecified rescue strategy, and the study was terminated for that patient. Because the primary outcome (colonoscopy success or failure) was definitively determined at the time of rescue, this patient was included in the PP analysis of the primary outcome as a treatment failure, in accordance with the principle that excluding treatment failures from the PP population in noninferiority trials would artificially inflate the success rate in the comparator group and bias the analysis in favor of noninferiority. In contrast, this patient was excluded from the PP analysis of secondary outcomes because study termination precluded the collection of complete follow-up data. The ITT population was primary for noninferiority testing, while secondary outcomes were analyzed in the PP population comprising patients who completed all prespecified study procedures and follow-up assessments.

PASS 15.0.5 (NCSS, LLC, Kesville, USA) was used for sample size calculations. Based on previous studies, the success rate of ciprofol was 99%,^3^ and the success rate of remimazolam was 97.5%,^23^ type I error rate α = 0.05 (one-sided), and the target power was 90%. The required sample size was 70 subjects per group, which was increased to 88 subjects per group for a 20% dropout rate for a total sample size of 176.

Noninferiority testing was adopted to compare the sedation efficacy of ciprofol and remimazolam for colonoscopy, with a prespecified absolute non-inferiority margin of 0.08.^24^^,^^25^ Clinically, an absolute difference ≤ 8% in colonoscopy completion success is considered negligible, without material influence on endoscopic workflow, patient safety, or routine clinical judgment. A 7%‒10% absolute non-inferiority margin for procedural success is commonly accepted in analogous gastrointestinal endoscopy sedation trials. The treatment difference was defined as the success rate in Group C minus that in Group R. A two-sided 95% Confidence Interval (95% CI) for the treatment difference was calculated using the Miettinen-Nurminen score method. Noninferiority was concluded if the lower bound of the two-sided 95% CI was above the prespecified noninferiority margin of -0.08.

SPSS 27.0 (Inc., Chicago, Illinois, USA) was used for statistical analysis. Normality was assessed via Shapiro-Wilk test and histograms; homogeneity of variance was tested by Levene’s test. Continuous data were compared by Student’s *t*-test or Mann-Whitney *U*-test and presented as mean ± Standard Deviation (SD) or median (Interquartile Range, IQR). Categorical data were analyzed using Pearson’s χ^2^, corrected χ^2^ or Fisher’s exact test. Bonferroni correction was applied to adjust for multiple comparisons among secondary outcomes, with a two-sided p-value < 0.0028 (0.05/18 secondary comparisons) considered statistically significant. Hemodynamic parameters (MAP, HR, and SpO_2_) were analyzed separately using repeated-measures analysis of variance, with group, time, and group-by-time interaction effects evaluated. A two-sided p-value < 0.05 was considered statistically significant for repeated-measures analyses.

## Results

From December 2023 to June 2024, a total of 350 patients were screened for outpatient colonoscopy at the Digestive Endoscopy Center of our hospital. A total of 174 patients were excluded due to non-compliance with the inclusion criteria. Ultimately, a total of 176 patients were included for randomization. In the ITT population (n = 176), 171 patients were included in the PP analysis of the primary outcome, and 170 patients were included in the PP analysis of secondary outcomes. In Group C, 2 patients were excluded because of loss to follow-up. In Group R, 2 patients were excluded because of loss to follow-up, and one because of withdrawal of informed consent. One patient who experienced sedation failure and received rescue propofol was included in the PP analysis of the primary outcome as a treatment failure, because the colonoscopy success/failure status was unequivocally assessable at the time of rescue; however, this patient was excluded from the PP analysis of secondary outcomes because study termination after rescue precluded the collection of complete follow-up data (Fig. 1). There were no significant differences in the baseline and demographic characteristics of patients between the two groups (Table 1).

**Figure 1:**
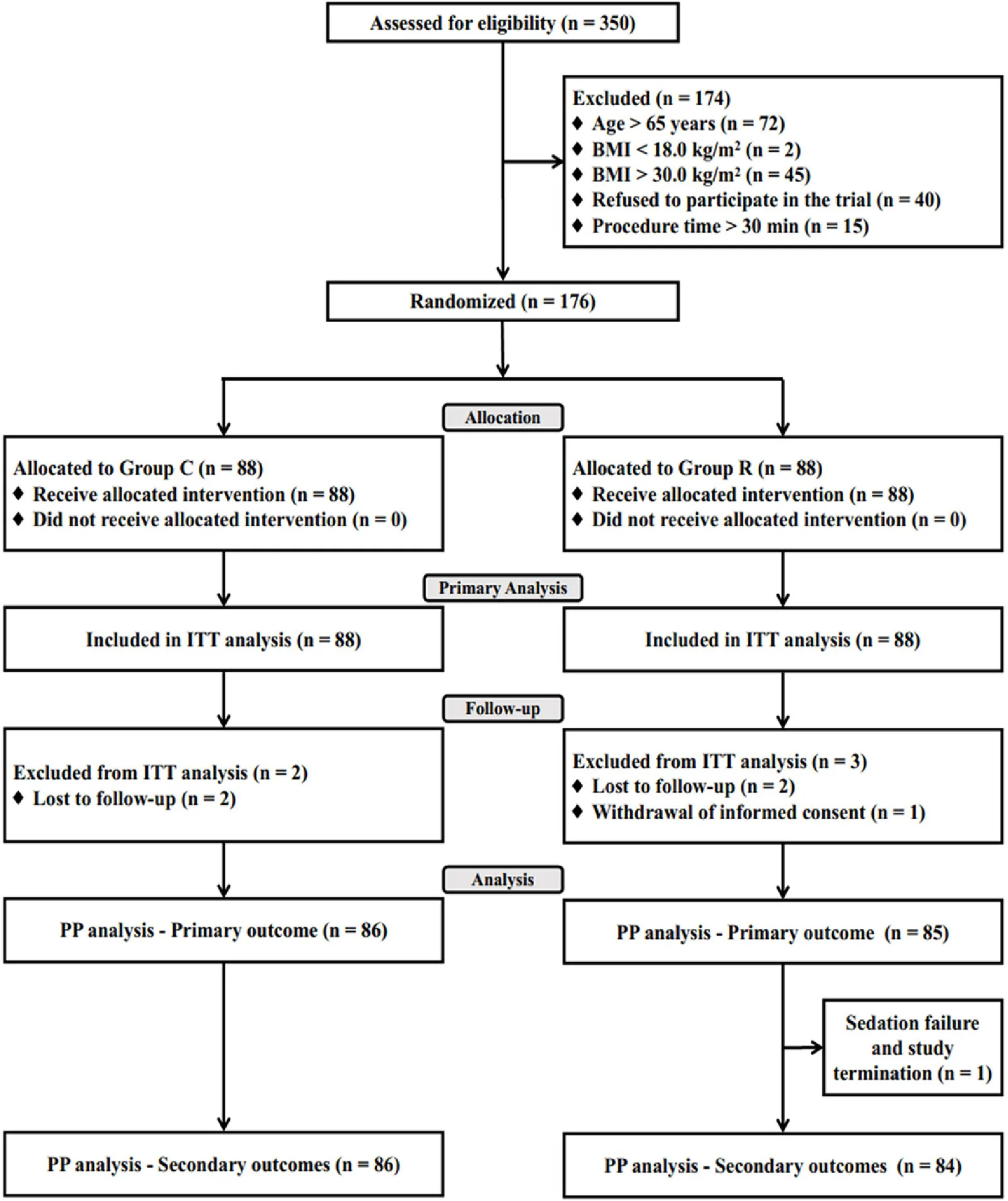
CONSORT flow diagram. CONSORT indicates Consolidated Standards of Reporting Trials. Group C, Ciprofol-Alfentanil group; Group R, Remimazolam-Alfentanil group; BMI, Body Mass Index; ITT, Intention-To-Treat; PP, Per-Protocol. One patient who received rescue propofol was included in the PP analysis of the primary outcome as a treatment failure, but was excluded from the PP analysis of secondary outcomes because study termination after rescue precluded the collection of complete follow-up data.

**Table 1:** Characteristics of the patients.

	Group C (n = 88)	Group R (n = 88)	p*-*value
**Age**^a^**, median (IQR); years**	44.0 (34.0‒54.0)	48.0 (40.3‒54.0)	0.103
**Sex**^b^**, Male/Female, n (%)**	47 (53.4)/41 (46.6)	53 (60.2)/35 (39.8)	0.361
**Height**^a^**, median (IQR); m**	1.7 (1.6‒1.8)	1.7 (1.6‒1.7)	0.490
**Weight**^a^**, median (IQR); kg**	70 (60‒80)	70 (60‒75)	0.435
**BMI**^a^**, median (IQR); kg.m^-2^**	24.6 (22.0‒27.0)	23.8 (22.0‒26.0)	0.277
**ASA status**^b^**, n (%)**			
I	25/88 (28.4)	15/88 (17.0)	0.072
II	63/88 (71.6)	73/88 (83.0)	

A p-value < 0.05 was considered statistically significant. IQR, Interquartile Range (25^th^‒75^th^ percentiles); BMI, Body Mass Index; ASA, American Society of Anaesthesiologists; Group C, Ciprofol-Alfentanil group; Group R, Remimazolam-Alfentanil group.

a Mann-Whitney *U*-test.

b Pearson Chi-Square.

The primary noninferiority analysis was conducted in the ITT population (n = 176). The success rate was 100% (88/88) in Group C and 98.9% (87/88) in Group R, with a treatment difference (ciprofol minus remimazolam) of 0.011 (95% CI: -0.024 to 0.048) (Table 2). The lower bound of the two-sided 95% CI was -0.024, which was above the prespecified noninferiority margin of -0.08, confirming the noninferiority of ciprofol relative to remimazolam. In the PP analysis of the primary outcome (n = 171), the success rate was 100% (86/86) in Group C and 98.8% (84/85) in Group R. The treatment difference (ciprofol minus remimazolam) was 0.012 (95% CI: -0.026 to 0.054). The lower bound of the two-sided 95% CI (-0.026) remained above the prespecified noninferiority margin of -0.08, further confirming the noninferiority of ciprofol relative to remimazolam.

**Table 2:** Colonoscopy success rates based on Intention-To-Treat (ITT) analysis.

	Group C (n = 88)	Group R (n = 88)	Estimated difference (95% CI)
**Success rate of colonoscopy, n (%)**	88 (100.0)	87 (98.9)	0.011 (-0.024‒0.048)

Group C, Ciprofol-Alfentanil group; Group R, Remimazolam-Alfentanil group; ITT, Intention-To-Treat; CI, Confidence Interval. The treatment difference was calculated as the colonoscopy success rate in Group C minus that in Group R. The 95% CI was calculated using the Miettinen-Nurminen score method. Noninferiority was concluded because the lower bound of the two-sided 95% CI (-0.024) was above the prespecified noninferiority margin of -0.08. In the Per-Protocol (PP) population (n = 171), the colonoscopy success rate was 100% (86/86) in Group C and 98.8% (84/85) in Group R; the estimated treatment difference was 0.012 (95% CI: -0.026 to 0.054, Miettinen-Nurminen score method), with the lower bound also exceeding -0.08. The patient who received rescue propofol was included as a treatment failure in this PP analysis of the primary outcome.

For secondary outcomes, the PP population comprised 170 patients (86 in Group C and 84 in Group R), as the sedation failure patient lacked complete follow-up data. After Bonferroni correction for multiple comparisons (adjusted p = 0.05/18 = 0.0028), only two differences remained statistically significant. Discharge time was shorter in Group R (7.0 [5.0‒9.0] vs. 8.5 [6.0‒12.0] minutes, p = 0.002). The total sedation cost per patient was also significantly lower in Group R (Group C vs. Group R: 8.01 [6.97‒9.12] vs. 5.10 [4.54‒5.95] US Dollars, p < 0.001). While induction time was numerically shorter in Group R (140 [134‒147] vs. 144 [136‒153] seconds, p = 0.040), maintenance dose proportion was lower in Group R (Group C vs. Group R: 0.09/0.49 mg [18.4%] vs. 0.02/0.32 mg [6.2%], p = 0.009), and hiccups occurred more frequently in Group R (0/86 [0.0%] vs. 5/84 [6.0%], p = 0.025), these differences were no longer statistically significant after Bonferroni correction. The two groups showed no significant differences in other adverse events (Table 3), postoperative QoR-15 scores at days 1, 3, 5, or 7 (Table 4), alert time, or satisfaction levels reported by endoscopists and patients.

**Table 3:** Secondary outcomes during the study period.

	Group C (n = 86)	Group R (n = 84)	p-value
**Induction time**^b^**, median (IQR); seconds**	144 (136-153)	140 (134-147)	0.040
**Operation time**^b^**, median (IQR); min**	15.5 (12.0-19.0)	16.0 (12.0-22.5)	0.702
**Alert time**^b^**, median (IQR); min**	10.0 (8.0-13.0)	11.0 (8.0-14.8)	0.358
**Discharge time**^b^**, median (IQR); min**	8.5 (6.0-12.0)	7.0 (5.0-9.0)	0.002
**Endoscopist satisfaction**^a^**, mean ± SD; score**	9.94 ± 0.28	9.87 ± 0.43	0.211
**Subject satisfaction**^a^**, mean ± SD; score**	10.00 ± 0.00	9.99 ± 0.11	0.494
**Induction consumption dose**^c^**, n/total n (%); mg.kg^-1^**	0.40/0.49 (81.6)	0.30/0.32 (93.8)	0.009
**Maintenance dose**^c^**, n/total n (%); mg.kg^-1^**	0.09/0.49 (18.4)	0.02/0.32 (6.2)	
**Average cost of sedative per case**^b^**, median (IQR); US Dollars**	8.01 (6.97-9.12)	5.10 (4.54-5.95)	< 0.001
**Intraoperative adverse reactions**^c^**, n/total n (%)**			
Hypotension	55/86 (64.0)	44/84 (52.4)	0.126
Hypotension require intervention	4/86 (4.7)	1/84 (1.2)	0.182
Injection pain	1/86 (1.2)	7/84 (8.3)	0.065
Bradycardia	1/86 (1.2)	0/84 (0.0)	1.000
Tachycardia	0/86 (0.0)	2/84 (2.4)	0.476
Respiratory depression	1/86 (1.2)	6/84 (7.1)	0.115
Apnea	0/86 (0.0)	1/84 (1.2)	0.991
Hiccup	0/86 (0.0)	5/84 (6.0)	0.025
**Postoperative adverse reactions**^c^**, n/total n (%)**			
PONV	1/86 (1.2)	1/84 (1.2)	0.987
Abdominal pain	11/86 (12.8)	5/84 (6.0)	0.125
Intraoperative awareness	0/86 (0.0)	0/84 (0.0)	‒

A two-sided p-value < 0.0028 was considered statistically significant after Bonferroni correction for multiple comparisons (0.05/18 secondary comparisons). Data are presented for the PP population for secondary outcomes (n = 170), which excluded the patient who received rescue propofol due to lack of complete follow-up data. IQR, Interquartile Range (25^th^‒75^th^ percentiles); NRS, Numerical rating scale (range 0 to 10); PONV, Postoperative Nausea and Vomiting; SD, Standard Deviation; Group C, Ciprofol-Alfentanil group; Group R, Remimazolam-Alfentanil group.

a *t* test.

b Mann-Whitney *U* test.

c Pearson Chi-Square.

**Table 4:** Comparison of QoR-15 scores at different time points.

Time Points	Group C (n = 86)	Group R (n = 84)	p*-*value
QoR-15 scores on POD1^a^	147 (140‒150)	147 (138‒150)	0.485
QoR-15 scores on POD3^a^	150 (144‒150)	149 (140‒150)	0.332
QoR-15 scores on POD5^a^	150 (146‒150)	150 (143‒150)	0.297
QoR-15 scores on POD7^a^	150 (147‒150)	150 (146‒150)	0.994

Data presented as median (IQR).

A two-sided p-value < 0.0028 was considered statistically significant after Bonferroni correction for multiple comparisons (0.05/18 secondary comparisons). Data are presented for the PP population for secondary outcomes (n = 170). IQR, Interquartile Range (25^th^‒75^th^ percentiles); QoR-15, the Quality of Recovery-15; POD1, Postoperative day-1; POD3, Postoperative day-3; POD5, Postoperative day-5; POD7, Postoperative day-7; SD, Standard deviation; Group C, Ciprofol-Alfentanil group; Group R, Remimazolam-Alfentanil group.

a Mann-Whitney *U* test.

Comparisons of MAP, HR and SpO_2_ at all predefined intraoperative time points between the two groups are shown in Figure 2. A significant main effect of time was observed for MAP (*F* = 179.772, p < 0.001). MAP decreased markedly at T2 and gradually recovered from T2 to T5. The main group effect did not reach statistical significance (*F* = 3.274, p = 0.072); the estimated marginal mean MAP was 86.59 mmHg in Group C and 88.98 mmHg in Group R (mean difference = -2.397 mmHg, p = 0.072). A significant group × time interaction was detected (*F* = 3.038, p = 0.017), indicating different MAP trajectories between groups. Simple-effect comparisons revealed no significant intergroup differences at T1 (p = 0.931), T2 (p = 0.507) and T3 (p = 0.229). Nominally higher MAP values were observed in Group R at T4 (p = 0.013) and T5 (p = 0.004). A significant main effect of time was detected for HR (*F* = 9.480, p < 0.001). HR slightly increased from T1 to T2, declined to the nadir at T4, and rebounded at T5, with significant within-time pairwise differences at multiple time points. A significant between-group effect was observed for HR (*F* = 18.212, p < 0.001); the estimated marginal mean HR was 70.58 bpm in Group C and 76.93 bpm in Group R (mean difference = -6.347 bpm, p < 0.001). A significant group × time interaction was identified (*F* = 11.187, p < 0.001), showing divergent HR dynamic trends over time. No significant intergroup difference was found at T1 (p = 0.061). Nominally higher HR values were observed in Group R at T2 (p < 0.001), T3 (p < 0.001), T4 (p = 0.003), and T5 (p = 0.002).

**Figure 2:**
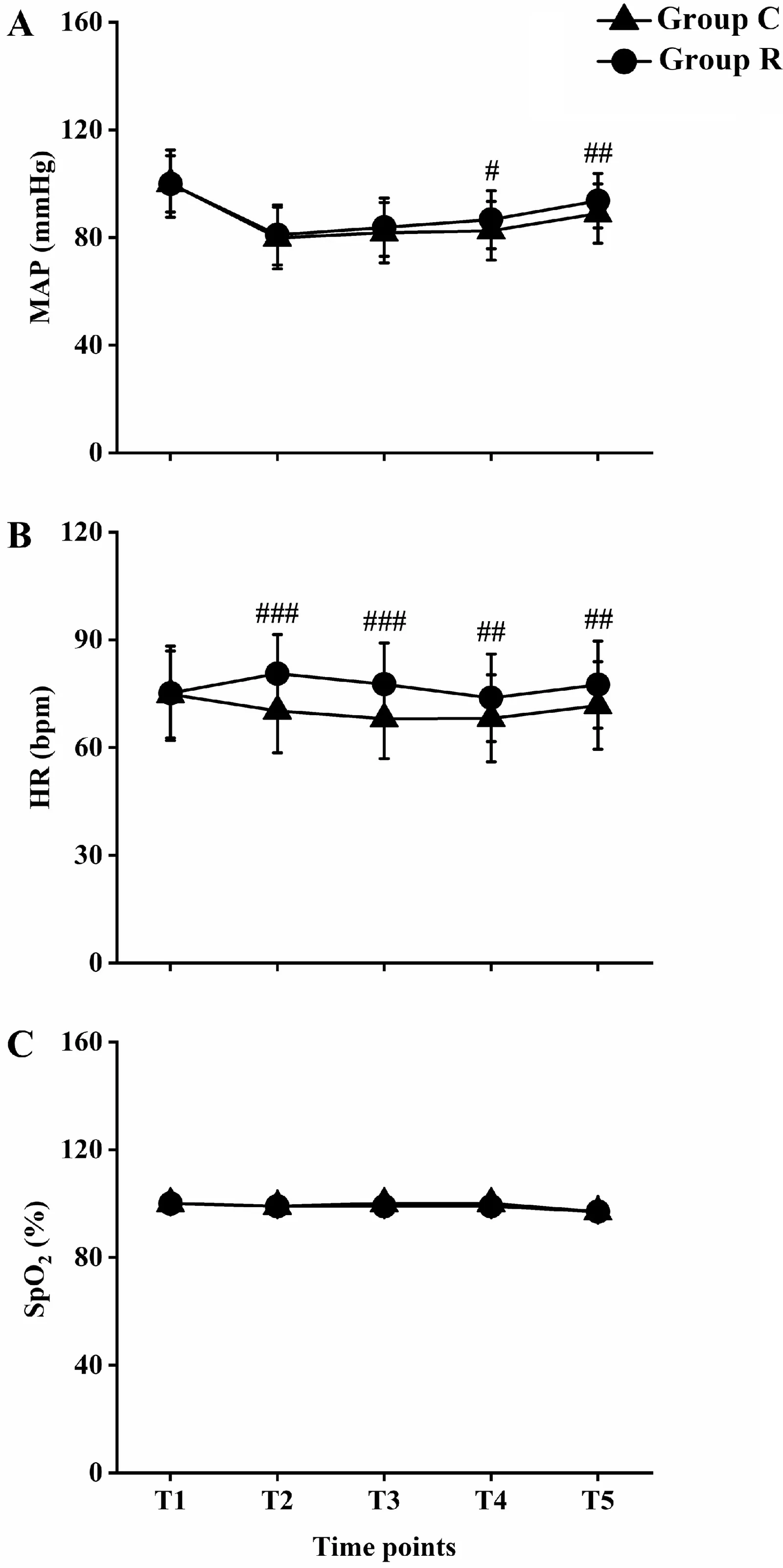
Comparison of MAP (A), HR (B) and SPO_2_ (C) between two groups. Group C, Ciprofol-Alfentanil group; Group R, Remimazolam-Alfentanil group; T1, upon entering the unit; T2, 1-minute after receiving an intravenous injection of ciprofol or remimazolam; T3, colonoscope reaching the ileocecal valve; T4, at the end of the procedure; T5, when leaving the room; MAP, Mean Arterial Pressure; HR, Heart Rate; SPO_2_, Pulse Oxygen Saturation. ^#^ p < 0.05, ^##^ p < 0.01, ^###^ p < 0.001.

## Discussion

This study compared the sedative effects of ciprofol versus remimazolam for colonoscopy sedation, accounting for potential opioid-related confounding. The results showed that both drugs offer favorable sedation, with safety profiles that appeared comparable within the limits of this study. The noninferiority of ciprofol relative to remimazolam was confirmed in both ITT and PP analyses of the primary outcome, with consistent conclusions from the two analytical datasets as recommended by non-inferiority trial reporting guidelines.

As a novel intravenous anesthetic, ciprofol has been gradually applied in colonoscopy sedation. A previous study investigated the optimal dose of ciprofol for colonoscopy procedures, and the results demonstrated that ciprofol at doses of 0.4 mg.kg^-1^ and 0.5 mg.kg^-1^ exhibited the most favorable benefit-risk profile.^9^ A study showed that the sedative effect of 0.4 mg.kg^-1^ ciprofol and 1.5 mg.kg^-1^ propofol in gastrointestinal endoscopy was equivalent.^3^ Consequently, our study selected ciprofol at a dose of 0.4 mg.kg^-1^ for inducing sedation.^9^ Remimazolam is often combined with opioids to meet the specific sedation requirements of endoscopic procedures.^11^^,^^22^^,^^26^ Previous studies have shown that a 0.3 mg.kg^-1^ dose of remimazolam provides a safe and effective anesthetic method for patients undergoing upper gastrointestinal endoscopy.^15^^,^^27^ Our study selected this dose for sedation induction. In our study, colonoscopy procedures using ciprofol or remimazolam, both combined with alfentanil, resulted in high rates of procedural success (100% and 98.9%, respectively). The impressive sedation success observed in both groups aligns with several earlier reports.^3^^,^^12^^,^^13^ It is noteworthy that the treatment difference in colonoscopy success rates between the ciprofol and remimazolam groups was 0.011 (1.1%) in the ITT population. The lower bound of the two-sided 95% CI for the treatment difference (ciprofol minus remimazolam) was -0.024, which was above the prespecified non-inferiority margin of -0.08. This statistical result definitively confirms that ciprofol met the predefined non-inferiority criterion compared to remimazolam for the colonoscopy success rate. Notably, one case of inadequate sedation occurred in the remimazolam group; this isolated finding is hypothesis-generating and does not allow general conclusions about remimazolam’s overall reliability.

Additionally, both groups demonstrated high success rates, which may be associated with the enhanced analgesic effect achieved by the combined use of alfentanil. Compared to other opioids, alfentanil has a faster onset time and shorter duration of action, making it more suitable for anesthesia in outpatient surgeries.^22^ Moreover, alfentanil effectively alleviated patient movements induced by intestinal traction during colonoscopy, thereby minimizing procedural interruptions caused by inadequate sedation.^28^ Ultimately, this, in combination with sedative drugs of different mechanisms of action, achieved a high success rate.

In our study, although remimazolam had a statistically shorter induction time than ciprofol, the absolute difference of only 4 seconds is too small to exert any practical impact on clinical practice. Meanwhile, the 1.5-minute reduction in discharge time, while not prominent for individual patients, could contribute to higher patient turnover and operational efficiency in high-volume endoscopy departments with a large daily caseload. This not only maximizes resource efficiency but also increases the procedural turnover rate per unit time.^29^ This study did not employ any antagonists, thus the recovery time of patients in both groups primarily depended on the metabolic process of the drugs themselves. To further shorten recovery time, consideration could be given to using the specific antagonist flumazenil in the remimazolam group.^30^ Meanwhile, in our study, remimazolam showed a lower proportion of maintenance dose relative to total consumption, indicating fewer intraoperative supplemental boluses and more stable sedation. This favorable profile may be attributed to its high receptor affinity. According to the sedative pricing at our center (ciprofol: 11.5 US dollars; remimazolam: 5.6 US dollars), remimazolam was associated with significantly lower drug acquisition cost. This finding should be interpreted cautiously, as the study was not powered for formal pharmacoeconomic analysis.

The two sedation regimens showed different hemodynamic trends and adverse event profiles during the procedure. The incidence of intraoperative hypotension was 64.0% in the ciprofol group and 52.4% in the remimazolam group. Notably, most hypotensive episodes were transient and mild, with spontaneous recovery shortly after occurrence. Only a small proportion of patients required vasopressor intervention, and no repeated or continuous vasopressor administration was needed in any patient. No clinical symptoms associated with hypotension were observed. The relatively high incidence of hypotension may be attributed to the synergistic effect on hemodynamics between sedative drugs and alfentanil, which is a common pharmacological interaction in endoscopic sedation regimens.^7^ Close intraoperative monitoring and timely symptomatic management ensured that these hemodynamic changes did not lead to adverse clinical outcomes, suggesting that both sedation regimens were generally well tolerated in this setting. Notably, five patients (5.95%) receiving remimazolam developed hiccups, consistent with prior reports.^10^ These episodes were mild and self-limited, with no examination interruption or severe adverse events.

This study has several limitations. Firstly, this single-center trial excluded elderly, obese and ASA III–IV high-risk patients, which reduces the generalizability of our results. Secondly, the sample size was calculated only for the primary endpoint, lacking adequate power to detect rare adverse events. Additionally, safety outcomes were analyzed in the PP population rather than the full treated population, as complete safety data were unavailable for 6 excluded patients. These factors should be considered when interpreting the safety findings. Thirdly, objective sedation depth monitoring was not performed. Sedation depth was assessed merely by clinical observation, which may ignore subtle intergroup differences in sedation stability. Finally, no propofol control group was set in this trial, making direct comparison with traditional propofol sedation unavailable. Further multi-arm trials containing propofol are required for more complete clinical evidence.

## Conclusion

In conclusion, this study demonstrated that ciprofol (0.4 mg.kg^-1^) and remimazolam (0.3 mg.kg^-1^), each combined with alfentanil (3 µg.kg^-1^), achieved adequate sedation for outpatient colonoscopy. Ciprofol was noninferior to remimazolam with respect to the colonoscopy success rate. Distinct hemodynamic profiles were seen across the two sedation regimens. Since this trial lacked power to capture rare adverse events, safety outcomes ought to be interpreted cautiously. The single sedation failure with remimazolam warrants further assessment within larger patient cohorts.

## Data availability statement

The fully de-identified individual participant raw data supporting the findings of this study are available in the Zenodo repository at https://doi.org/10.5281/zenodo.21625752. All personal identifiable information was completely removed to protect patient confidentiality.

## Ethics statement

The study was conducted following the guidelines of the Declaration of Helsinki. The research project for this study was submitted and approved by the Ethics Committee of the People’s Hospital of Liaoning Province on September 28, 2022 (approval number: 2022 H002) and was registered in the clinical registration center under number ChiCTR2300070996 on April 28, 2023 (https://www.chictr.org.cn/showproj.html?proj=195336). Written informed consent was obtained from all subjects participating in the trial.

## Declaration of generative AI and AI-assisted technologies

This study did not utilize generative AI or AI-assisted technologies.

## Authorship contribution statement

Xuan Liu: Conceptualization; data curation; formal analysis; investigation; writing-original draft.

Qianqian Tang: Data curation; methodology; writing-original draft.

Ning Zou: Data curation; investigation.

Changming Wang: Conceptualization; methodology; project administration; resources, supervision, validation; visualization; writing-review & editing.

Xuejun Guo: Project administration; resources; supervision.

## Funding

This work was supported by the Xingliao Talent Program Project [grant number XLYC2412057] and the Research and Development Fund Project for Medical and Health Undertakings [grant number S231], and the China International Science Exchange Foundation [grant number SWYY2025001007]. The funders had no role in study design, data collection, and analysis, preparation of the manuscript, or decision to publish.

## Conflicts of interest

The authors declare no conflicts of interest.
